# Supplementary material for: Avian influenza viruses in wild birds in Canada following incursions of highly pathogenic H5N1 virus from Eurasia in 2021–2022
Source: mBio. 2024 Jul 16;15(8):e03203-23. doi: 10.1128/mbio.03203-23 (PMC11323545; doi:10.1128/mbio.03203-23)
Supplement: Caption — Supplemental movie caption. [file mbio.03203-23-s0002.docx]

**Other Supplemental Materials Headings**

**Video 1**. Time series of sick and dead wild birds confirmed to be highly pathogenic avian influenza virus (HPAIV) positive in Canada between November 2021 and December 2022. Taxonomic grouping represented by colored symbology.
