# Supplementary material for: Avian influenza viruses in wild birds in Canada following incursions of highly pathogenic H5N1 virus from Eurasia in 2021–2022
Source: mBio. 2024 Jul 16;15(8):e03203-23. doi: 10.1128/mbio.03203-23 (PMC11323545; doi:10.1128/mbio.03203-23)
Supplement: Supplemental Tables — Tables S1 and S2. [file mbio.03203-23-s0003.docx]

**Supplemental Tables**

Table S1. Live and hunter-harvested and sick and dead wild birds submitted for testing and suspect or confirmed highly pathogenic avian influenza virus (HPAIV) or low pathogenicity avian influenza virus (LPAIV) positive in Canada between November 2021 – November 2022.

| **Taxonomic Order** | **Taxonomic Family** | **Species Group** | **Common Name** | **Scientific Name** | **Live/Harvest** | |  | **Morbidity/Mortality** | |  |
| --- | --- | --- | --- | --- | --- | --- | --- | --- | --- | --- |
|  |  |  |  |  |  | |  |  | |  |
|  |  |  |  |  | **Suspect/**  **Confirmed HPAIV** | **Suspect/**  **Confirmed LPAIV** | **Total** | **Suspect/ Confirmed HPAIV^a^** | **Suspect/**  **Confirmed LPAIV** | **Total** |
| **Accipitriformes** | **Accipitridae**  **(Hawks, Eagles, Kites)** | **Eagles** | Bald Eagle | *Haliaeetus leucocephalus* | - | - | **-** | 103 (34.7%) | 5 (1.7%) | **297** |
|  |  |  | Golden Eagle | *Aquila chrysaetos* | - | - | **-** | 0 | 0 | **6** |
|  |  | **Hawks** | Broad-winged Hawk | *Buteo platypterus* | - | - | **-** | 2 (16.7%) | 0 | **12** |
|  |  |  | Cooper's Hawk | *Accipiter cooperii* | - | - | **-** | 9 (13.2%) | 0 | **68** |
|  |  |  | Northern Goshawk | *Accipiter gentilis* | - | - | **-** | 0 | 0 | **13** |
|  |  |  | Northern Harrier | *Circus hudsonius* | - | - | **-** | 0 | 0 | **3** |
|  |  |  | Red-shouldered Hawk | *Buteo lineatus* | - | - | **-** | 1 (100.0%) | 0 | **1** |
|  |  |  | Red-tailed Hawk | *Buteo jamaicensis* | - | - | **-** | 78 (42.2%) | 6 (3.2%) | **185** |
|  |  |  | Rough-legged Hawk | *Buteo lagopus* | - | - | **-** | 21 (72.4%) | 0 | **29** |
|  |  |  | Sharp-shinned Hawk | *Accipiter striatus* | - | - | **-** | 0 | 1 (2.4%) | **41** |
|  |  |  | Swainson's Hawk | *Buteo swainsoni* | - | - | **-** | 9 (52.9%) | 0 | **17** |
|  |  |  | Unidentified Accipiter Hawk | *-* | - | - | **-** | 0 | 0 | **1** |
|  | **Cathartidae**  **(New World Vultures)** | **Other** | Turkey Vulture | *Cathartes aura* | - | - | **-** | 65 (76.5%) | 1 (1.2%) | **85** |
|  | **Pandionidae**  **(Osprey)** |  | Osprey | *Pandion haliaetus* | - | - | **-** | 0 | 0 | **15** |
| **Total Accipitriformes** |  |  |  |  | **-** | **-** | **-** | **288 (37.3%)** | **13 (1.7%)** | **773** |
| **Anseriformes** | **Anatidae**  **(Ducks, Geese Waterfowl)** | **Dabbling ducks** | American Black Duck | *Anas rubripes* | 48 (13.3%) | 110 (30.6%) | **360** | 2 (11.8%) | 2 (11.8%) | **17** |
|  |  |  | American Wigeon | *Mareca americana* | 3 (3.5%) | 3 (3.5%) | **85** | 2 (50.0%) | 0 | **4** |
|  |  |  | Blue-winged Teal | *Spatula discors* | 18 (2.2%) | 231 (28.5%) | **811** | 9 (60.0%) | 0 | **15** |
|  |  |  | Cinnamon Teal | *Spatula cyanoptera* | 1 (100.0%) | 0 | **1** | - | - | **-** |
|  |  |  | Eurasian Wigeon | *Mareca penelope* | 0 | 0 | **2** | - | - | **-** |
|  |  |  | Gadwall | *Mareca strepera* | 0 | 1 (12.5%) | **8** | 2 (50.0%) | 0 | **4** |
|  |  |  | Green-winged Teal | *Anas crecca* | 38 (7.3%) | 107 (20.7%) | **518** | 11 (78.6%) | 0 | **14** |
|  |  |  | Mallard | *Anas platyrhynchos* | 269 (10.0%) | 511 (19.1%) | **2677** | 22 (11.4%) | 2 (1.0%) | **193** |
|  |  |  | Mallard X American Black Duck | *Anas platyrhynchos x rubripes* | 5 (14.3%) | 5 (14.3%) | **35** | - | - | **-** |
|  |  |  | Northern Pintail | *Anas acuta* | 21 (11.4%) | 34 (18.5%) | **184** | 2 (50.0%) | 0 | **4** |
|  |  |  | Northern Shoveler | *Spatula clypeata* | 0 | 5 (41.7%) | **12** | 3 (75.0%) | 1 (25.0%) | **4** |
|  |  |  | Wood Duck | *Aix sponsa* | 7 (4.1%) | 29 (17.0%) | **171** | 12 (57.1%) | 0 | **21** |
|  |  |  | Unidentified Duck | - | 2 (8.0%) | 4 (19.1%) | **21** | 2 (28.6%) | 0 | **7** |
|  |  | **Diving ducks and seaducks** | Black Scoter | *Melanitta americana* | 0 | 0 | **5** | 1 (33.3%) | 0 | **3** |
|  |  |  | Bufflehead | *Bucephala albeola* | 0 | 1 (2.9%) | **35** | 1 (20.0%) | 0 | **5** |
|  |  |  | Canvasback | *Aythya valisineria* | 7 (19.4%) | 2 (5.6%) | **36** | 1 (50.0%) | 0 | **2** |
|  |  |  | Common Eider | *Somateria mollissima* | 0 | 1 (0.4%) | **272** | 60 (62.5%) | 1 (1.0%) | **96** |
|  |  |  | Common Goldeneye | *Bucephala clangula* | 0 | 1 (12.5%) | **8** | 1 (10.0%) | 1 (10.0%) | **10** |
|  |  |  | Common Merganser | *Mergus merganser* | - | - | **-** | 2 (28.6%) | 0 | **7** |
|  |  |  | Greater Scaup | *Aythya marila* | - | - | **-** | 0 | 0 | **1** |
|  |  |  | Hooded Merganser | *Lophodytes cucullatus* | 0 | 0 | **2** | 3 (33.3%) | 0 | **9** |
|  |  |  | King Eider | *Somateria spectabilis* | 0 | 0 | **4** | - | - | **-** |
|  |  |  | Lesser Scaup | *Aythya affinis* | 0 | 0 | **18** | 0 | 0 | **5** |
|  |  |  | Long-tailed Duck | *Clangula hyemalis* | - | - | **-** | 1 (4.2%) | 0 | **24** |
|  |  |  | Red-breasted Merganser | *Mergus serrator* | - | - | **-** | 4 (33.3%) | 0 | **12** |
|  |  |  | Redhead | *Aythya americana* | 2 (2.0%) | 0 | **101** | 1 (16.7%) | 0 | **6** |
|  |  |  | Ring-necked Duck | *Aythya collaris* | 0 | 1 (0.7%) | **155** | - | - | **-** |
|  |  |  | Ruddy Duck | *Oxyura jamaicensis* | - | - | **-** | 0 | 0 | **5** |
|  |  |  | Surf Scoter | *Melanitta perspicillata* | 0 | 2 (20.0%) | **10** | 1 (12.5%) | 0 | **8** |
|  |  |  | Tufted Duck | *Aythya fuligula* | - | - | **-** | 0 | 0 | **2** |
|  |  |  | White-winged Scoter | *Melanitta deglandi* | 0 | 1 (4.2%) | **24** | 0 | 0 | **4** |
|  |  |  | Unidentified Scaup | - | 0 | 1 (16.7%) | **6** | - | - | **-** |
|  |  | **Geese** | Atlantic Brant | *Branta bernicla hrota* | 0 | 0 | **160** | - | - | **-** |
|  |  |  | Black Brant | *Branta bernicla nigricans* | 0 | 0 | **43** | 1 (50.0%) | 0 | **2** |
|  |  |  | Cackling Goose | *Branta hutchinsii* | 0 | 0 | **11** | 13 (72.2%) | 0 | **18** |
|  |  |  | Canada Goose | *Branta canadensis* | 1 (0.1%) | 3 (0.2%) | **1427** | 248 (47.4%) | 3 (0.6%) | **523** |
|  |  |  | Greater White-fronted Goose | *Anser albifrons* | 0 | 0 | **6** | 0 | 0 | **10** |
|  |  |  | Ross's Goose | *Anser rossii* | 0 | 0 | **16** | 13 (81.3%) | 2 (12.5%) | **16** |
|  |  |  | Snow Goose | *Anser caerulescens* | 125 (5.1%) | 84 (3.4%) | **2475** | 152 (81.3%) | 1 (0.5%) | **187** |
|  |  | **Swans** | Mute Swan | *Cygnus olor* | 0 | 1 (100.0%) | **1** | 0 | 0 | **18** |
|  |  |  | Trumpeter Swan | *Cygnus buccinator* | - | - | **-** | 13 (21.3%) | 0 | **61** |
|  |  |  | Tundra Swan | *Cygnus columbianus* | - | - | **-** | 6 (66.7%) | 0 | **9** |
| **Total Anseriformes** |  |  |  |  | **547 (5.6%)** | **1138 (11.7%)** | **9700** | **589 (44.4%)** | **13 (1.0%)** | **1326** |
| **Apodiformes** | **Trochilidae (Hummingbirds)** |  | Anna's Hummingbird | *Calypte anna* | - | - | **-** | 0 | 0 | **2** |
|  |  |  | Ruby-throated Hummingbird | *Archilochus colubris* | - | - | **-** | 0 | 0 | **4** |
|  |  |  | Rufous Hummingbird | *Selasphorus rufus* | - | - | **-** | 0 | 0 | **5** |
| **Total Apodiformes** |  |  |  |  | **-** | **-** | **-** | **0** | **0** | **11** |
| **Caprimulgiformes** | **Caprimulgidae (Nightjars and Allies)** |  | Common Nighthawk | *Chordeiles minor* | - | - | **-** | 0 | 0 | **3** |
| **Total Caprimulgiformes** |  |  |  |  | **-** | **-** | **-** | **0** | **0** | **3** |
| **Charadriiformes** | **Alcidae**  **(Auks, Murres, Puffins)** |  | Ancient Murrelet | *Synthliboramphus antiquus* | - | - | **-** | 0 | 0 | **1** |
|  |  |  | Atlantic Puffin | *Fratercula arctica* | 1 (1.6%) | 0 | **64** | 5 (20.0%) | 0 | **25** |
|  |  |  | Black Guillemot | *Cepphus grylle* | 0 | 0 | **69** | 0 | 0 | **2** |
|  |  |  | Common Murre | *Uria aalge* | 26 (61.9%) | 0 | **42** | 48 (59.3%) | 1 (1.2%) | **81** |
|  |  |  | Dovekie | *Alle alle* | - | - | **-** | 3 (12.0%) | 0 | **25** |
|  |  |  | Marbeled Murrelet | *Brachyramphus marmoratus* | - | - | **-** | 0 | 0 | **2** |
|  |  |  | Razorbill | *Alca torda* | - | - | **-** | 9 (47.4%) | 0 | **19** |
|  |  |  | Rhinoceros Auklet | *Cerorhinca monocerata* | - | - | **-** | 0 | 0 | **1** |
|  |  |  | Thick-billed Murre | *Uria lomvia* | 1 (0.6%) | 19 (10.9%) | **174** | 1 (1.5%) | 1 (1.5%) | **65** |
|  | **Charadriidae**  **(Plovers, Lapwings)** |  | Killdeer | *Charadrius vociferus* | - | - | **-** | 0 | 0 | **1** |
|  |  |  | Piping Plover | *Charadrius melodus* | - | - | **-** | 0 | 0 | **2** |
|  |  |  | Semipalmated Plover | *Charadrius semipalmatus* | 0 | 0 | **7** | - | - | **-** |
|  | **Laridae**  **(Gulls, Terns, Skimmers)** |  | Arctic Tern | *Sterna paradisaea* | 0 | 0 | **16** | 2 (8.7%) | 0 | **23** |
|  |  |  | Black Tern | *Chlidonias niger* | 0 | 0 | **16** | - | - | **-** |
|  |  |  | Black-legged Kittiwake | *Rissa tridactyla* | 5 (12.8%) | 0 | **39** | 10 (76.9%) | 1 (7.7%) | **13** |
|  |  |  | California Gull | *Larus californicus* | - | - | **-** | 4 (25.0%) | 2 (12.5%) | **16** |
|  |  |  | Common Tern | *Sterna hirundo* | 0 | 0 | **20** | 6 (14.6%) | 0 | **41** |
|  |  |  | Franklin's Gull | *Leucophaeus pipixcan* | - | - | **-** | 2 (13.3%) | 2 (13.3%) | **15** |
|  |  |  | Glaucous Gull | *Larus hyperboreus* | 0 | 0 | **1** | 1 (50.0%) | 0 | **2** |
|  |  |  | Glaucous-winged Gull | *Larus glaucescens* | - | - | **-** | 5 (14.7%) | 0 | **34** |
|  |  |  | Gray Gull | *Leucophaeus modestus* | - | - | **-** | 0 | 0 | **1** |
|  |  |  | Great Black-backed Gull | *Larus marinus* | 0 | 0 | **13** | 43 (42.2%) | 1 (1.0%) | **102** |
|  |  |  | Herring Gull | *Larus argentatus* | 2 (3.0%) | 0 | **66** | 46 (25.8%) | 4 (2.3%) | **178** |
|  |  |  | Iceland Gull | *Larus glaucoides* | 0 | 0 | **24** | 2 (22.2%) | 0 | **9** |
|  |  |  | Lesser Black-backed Gull | *Larus fuscus* | - | - | **-** | 0 | 0 | **1** |
|  |  |  | Ring-billed Gull | *Larus delawarensis* | 0 | 1 (0.5%) | **200** | 14 (10.0%) | 9 (6.4%) | **140** |
|  |  |  | Roseate Tern | *Sterna dougallii* | 0 | 0 | **2** | 0 | 0 | **1** |
|  | **Unidentified Charadriiformes** |  | Unidentified Larus Gull | *-* | - | - | **-** | 4 (12.5%) | 0 | **32** |
|  | **Recurvirostridae**  **(Stilts, Avocets)** |  | American Avocet | *Recurvirostra americana* | - | - | **-** | 0 | 0 | **1** |
|  |  |  | Black-necked Stilt | *Himantopus mexicanus* | - | - | **-** | 0 | 0 | **1** |
|  | **Scolopacidae (Sandpipers, Allies)** |  | American Woodcock | *Scolopax minor* | - | - | **-** | 0 | 0 | **9** |
|  |  |  | Dunlin | *Calidris alpina* | 0 | 0 | **23** | - | - | **-** |
|  |  |  | Greater Yellowlegs | *Tringa melanoleuca* | - | - | **-** | 1 (100.0%) | 0 | **1** |
|  |  |  | Least Sandpiper | *Calidris minutilla* | 0 | 0 | **1** | - | - | **-** |
|  |  |  | Pectoral Sandpiper | *Calidris melanotos* | - | - | **-** | 0 | 0 | **1** |
|  |  |  | Red Knot | *Calidris canutus* | 0 | 0 | **38** | - | - | **-** |
|  |  |  | Red-necked Phalarope | *Phalaropus lobatus* | - | - | **-** | 0 | 0 | **1** |
|  |  |  | Semipalmated Sandpiper | *Calidris pusilla* | 0 | 0 | **26** | 0 | 0 | **2** |
|  |  |  | White-rumped Sandpiper | *Calidris fuscicollis* | 0 | 0 | **37** | - | - | **-** |
|  |  |  | Willet | *Tringa semipalmata* | - | - | **-** | 1 (100.0%) | 0 | **1** |
|  | **Stercorariidae**  **(Skuas, Jaegers)** |  | Pomarine Jaeger | *Stercorarius pomarinus* | - | - | **-** | 0 | 0 | **1** |
| **Total Charadriiformes** |  |  |  |  | **35 (4.0%)** | **20 (2.3%)** | **878** | **207 (24.4%)** | **21 (2.5%)** | **850** |
| **Columbiformes** | **Columbidae**  **(Pigeons, Doves)** |  | Eurasian Collared Dove | *Streptopelia decaocto* | - | - | **-** | 0 | 0 | **7** |
|  |  |  | Mourning Dove | *Zenaida macroura* | - | - | **-** | 0 | 0 | **29** |
|  |  |  | Rock Pigeon | *Columba livia* | 0 | 0 | **2** | 3 (1.8%)^b^ | 1 (0.60%) | **166** |
| **Total Columbiformes** |  |  |  |  | **0** | **0** | **2** | **3 (1.5%)** | **1 (0.5%)** | **202** |
| **Coraciiformes** | **Alcedinidae (Kingfishers)** |  | Belted Kingfisher | *Megaceryle alcyon* | - | - | **-** | 0 | 0 | **6** |
| **Total Coraciiformes** |  |  |  |  | **-** | **-** | **-** | **0** | **0** | **6** |
| **Falconiformes** | **Falconidae**  **(Falcons, Caracaras)** |  | American Kestrel | *Falco sparverius* | - | - | **-** | 0 | 0 | **8** |
|  |  |  | Merlin | *Falco columbarius* | - | - | **-** | 0 | 0 | **81** |
|  |  |  | Peregrine Falcon | *Falco peregrinus* | - | - | **-** | 22 (56.4%) | 0 | **39** |
| **Total Falconiformes** |  |  |  |  | **-** | **-** | **-** | **18 (14.52%)** | **0** | **124** |
| **Galliformes** | **Odontophoridae**  **(New World Quail)** |  | Northern Bobwhite | *Colinus virginianus* | - | - | **-** | 0 | 0 | **1** |
|  | **Phasianidae (Pheasants, Grouse, Allies)** |  | Gray Partridge | *Perdix perdix* | - | - | **-** | 0 | 0 | **5** |
|  |  |  | Greater Sage-grouse | *Centrocercus urophasianus* | - | - | **-** | 0 | 0 | **1** |
|  |  |  | Ring-necked Pheasant | *Phasianus colchicus* | - | - | **-** | 1 (2.9%) | 0 | **35** |
|  |  |  | Rock Ptarmigan | *Lagopus muta* | - | - | **-** | 0 | 0 | **2** |
|  |  |  | Ruffed Grouse | *Bonasa umbellus* | - | - | **-** | 2 (2.9%) | 0 | **69** |
|  |  |  | Sharp-tailed Grouse | *Tympanuchus phasianellus* | - | - | **-** | 0 | 0 | **2** |
|  |  |  | Spruce Grouse | *Canachites canadensis* | - | - | **-** | 0 | 0 | **2** |
|  |  |  | Wild Turkey | *Meleagris gallopavo* | - | - | **-** | 2 (8.7%)^c^ | 0 | **23** |
|  | **Unidentified Galliformes** |  | Unidentified Phasianidae | *-* | - | - | **-** | 0 | 0 | **3** |
| **Total Galliformes** |  |  |  |  | **-** | **-** | **-** | **5 (3.5%)** | **0** | **143** |
| **Gaviiformes** | **Gaviidae**  **(Loons)** |  | Common Loon | *Gavia immer* | - | - | **-** | 0 | 0 | **36** |
|  |  |  | Red-throated Loon | *Gavia stellata* | - | - | **-** | 0 | 0 | **3** |
| **Total Gaviiformes** |  |  |  |  | **-** | **-** | **-** | **0** | **0** | **39** |
| **Gruiformes** | **Gruidae**  **(Cranes)** |  | Sandhill Crane | *Antigone canadensis* | - | - | **-** | 0 | 0 | **2** |
|  |  |  | Whooping Crane | *Grus americana* | 0 | 0 | **15** | 0 | 0 | **1** |
|  | **Rallidae**  **(Rails, Gallinules, Coots)** |  | American Coot | *Fulica americana* | 0 | 2 (3.1%) | **64** | 0 | 1 (14.3%) | **7** |
|  |  |  | Common Moorhen | *Gallinula chloropus* | - | - | **-** | 0 | 0 | **1** |
| **Total Gruiformes** |  |  |  |  | **0** | **2 (2.5%)** | **79** | **-** | **1 (9.1%)** | **11** |
| **Passeriformes** | **Corvidae**  **(Crows, Jays, Magpies)** | **Corvids** | American Crow | *Corvus brachyrhynchos* | - | - | **-** | 136 (17.5%) | 4 (0.5%) | **776** |
|  |  |  | Black-billed Magpie | *Pica hudsonia* | - | - | **-** | 39 (30.2%) | 0 | **129** |
|  |  |  | Blue Jay | *Cyanocitta cristata* | - | - | **-** | 4 (4.9%) | 0 | **81** |
|  |  |  | Common Raven | *Corvus corax* | - | - | **-** | 41 (29.5%) | 0 | **139** |
|  |  |  | Steller’s Jay | *Cyanocitta stelleri* | - | - | **-** | 0 | 0 | **1** |
|  | **Alaudidae**  **(Larks)** | **Other** | Horned Lark | *Eremophila alpestris* | - | - | **-** | 0 | 0 | **1** |
|  | **Bombycillidae (Waxwings)** |  | Bohemian Waxwing | *Bombycilla garrulus* | - | - | **-** | 1 (4.6%) | 0 | **22** |
|  |  |  | Cedar Waxwing | *Bombycilla cedrorum* | - | - | **-** | 0 | 0 | **15** |
|  | **Calcariidae**  **(Longspurs, Snow Buntings)** |  | Chestnut-collared Longspur | *Calcarius ornatus* | - | - | **-** | 0 | 0 | **1** |
|  |  |  | Snow Bunting | *Plectrophenax nivalis* | - | - | **-** | 0 | 0 | **1** |
|  | **Cardinalidae**  **(Cardinals, Allies)** |  | Northern Cardinal | *Cardinalis cardinalis* | - | - | **-** | 0 | 0 | **5** |
|  |  |  | Rose-breasted Grosbeak | *Pheucticus ludovicianus* | - | - | **-** | 0 | 0 | **2** |
|  | **Fringillidae**  **(Finches, Euphonias, Allies)** |  | American Goldfinch | *Spinus tristis* | - | - | **-** | 0 | 0 | **13** |
|  |  |  | Common Redpoll | *Acanthis flammea* | - | - | **-** | 0 | 0 | **23** |
|  |  |  | House Finch | *Haemorhous mexicanus* | - | - | **-** | 0 | 0 | **12** |
|  |  |  | Pine Grosbeak | *Pinicola enucleator* | - | - | **-** | 0 | 0 | **1** |
|  |  |  | Pine Siskin | *Spinus pinus* | - | - | **-** | 0 | 0 | **21** |
|  |  |  | Purple Finch | *Haemorhous purpureus* | - | - | **-** | 0 | 0 | **5** |
|  |  |  | Red Crossbill | *Loxia curvirostra* | - | - | **-** | 0 | 0 | **3** |
|  |  |  | White-winged Crossbill | *Loxia leucoptera* | - | - | **-** | 0 | 0 | **3** |
|  | **Hirundinidae (Swallows)** |  | Barn Swallow | *Hirundo rustica* | - | - | **-** | 0 | 0 | **8** |
|  |  |  | Cliff Swallow | *Petrochelidon pyrrhonota* | - | - | **-** | 0 | 0 | **1** |
|  |  |  | Purple Martin | *Progne subis* | - | - | **-** | 0 | 0 | **11** |
|  |  |  | Tree Swallow | *Tachycineta bicolor* | - | - | **-** | 0 | 0 | **9** |
|  | **Icteridae**  **(Troupials, Allies)** |  | Baltimore Oriole | *Icterus galbula* | - | - | **-** | 0 | 0 | **1** |
|  |  |  | Brewer's Blackbird | *Euphagus cyanocephalus* | - | - | **-** | 0 | 0 | **2** |
|  |  |  | Brown-headed Cowbird | *Molothrus ater* | - | - | **-** | 0 | 0 | **4** |
|  |  |  | Common Grackle | *Quiscalus quiscula* | - | - | **-** | 2 (4.4%) | 2 (4.4%) | **45** |
|  |  |  | Red-winged Blackbird | *Agelaius phoeniceus* | - | - | **-** | 0 | 0 | **4** |
|  | **Laniidae**  **(Shrikes)** |  | Loggerhead Shrike | *Lanius ludovicianus* | - | - | **-** | 0 | 0 | **12** |
|  |  |  | Northern Shrike | *Lanius borealis* | - | - | **-** | 0 | 0 | **1** |
|  | **Mimidae**  **(Mockingbirds, Thrashers)** |  | Gray Catbird | *Dumetella carolinensis* | - | - | **-** | 0 | 0 | **1** |
|  | **Paridae**  **(Tits, Chickadees, Titmice)** |  | Black-capped Chickadee | *Poecile atricapillus* | - | - | **-** | 0 | 0 | **25** |
|  | **Parulidae**  **(New World Warblers)** |  | Bay-breasted Warbler | *Setophaga castanea* | - | - | **-** | 0 | 0 | **3** |
|  |  |  | Magnolia Warbler | *Setophaga magnolia* | - | - | **-** | 0 | 0 | **1** |
|  |  |  | Northern Waterthrush | *Parkesia noveboracensis* | - | - | **-** | 0 | 0 | **2** |
|  |  |  | Orange-crowned Warbler | *Leiothlypis celata* | - | - | **-** | 0 | 0 | **2** |
|  |  |  | Tennessee Warbler | *Leiothlypis peregrina* | - | - | **-** | 0 | 0 | **2** |
|  |  |  | Wilson's Warbler | *Cardellina pusilla* | - | - | **-** | 0 | 0 | **1** |
|  |  |  | Yellow Warbler | *Setophaga petechia* | - | - | **-** | 0 | 0 | **2** |
|  |  |  | Yellow-rumped Warbler | *Setophaga coronata* | - | - | **-** | 0 | 0 | **7** |
|  | **Passerellidae**  **(New World Sparrows)** |  | American Tree Sparrow | *Spizelloides arborea* | - | - | **-** | 0 | 0 | **5** |
|  |  |  | Chipping Sparrow | *Spizella passerina* | - | - | **-** | 0 | 0 | **6** |
|  |  |  | Dark-eyed Junco | *Junco hyemalis* | - | - | **-** | 0 | 0 | **17** |
|  |  |  | Fox Sparrow | *Passerella iliaca* | - | - | **-** | 0 | 0 | **1** |
|  |  |  | Song Sparrow | *Melospiza melodia* | - | - | **-** | 0 | 0 | **1** |
|  |  |  | Spotted Towhee | *Pipilo maculatus* | - | - | **-** | 0 | 0 | **2** |
|  |  |  | Swamp Sparrow | *Melospiza georgiana* | - | - | **-** | 0 | 0 | **1** |
|  |  |  | White-crowned Sparrow | *Zonotrichia leucophrys* | - | - | **-** | 0 | 0 | **4** |
|  |  |  | White-throated Sparrow | *Zonotrichia albicollis* | - | - | **-** | 0 | 0 | **7** |
|  | **Passeridae**  **(Old World Sparrows)** |  | House Sparrow | *Passer domesticus* | - | - | **-** | 1 (2.3%)^d^ | 0 | **44** |
|  | **Sittidae**  **(Nuthatches)** |  | Red-breasted Nuthatch | *Sitta canadensis* | - | - | **-** | 0 | 0 | **1** |
|  |  |  | White-breasted Nuthatch | *Sitta carolinensis* | - | - | **-** | 0 | 0 | **3** |
|  | **Sturnidae**  **(Starlings)** |  | European Starling | *Sturnus vulgaris* | - | - | **-** | 0 | 2 (6.3%) | **32** |
|  | **Turdidae**  **(Thrushes, Allies)** |  | American Robin | *Turdus migratorius* | - | - | **-** | 0 | 0 | **171** |
|  |  |  | Eastern Bluebird | *Sialia sialis* | - | - | **-** | 0 | 0 | **1** |
|  |  |  | Hermit Thrush | *Catharus guttatus* | - | - | **-** | 0 | 0 | **2** |
|  |  |  | Swainson's Thrush | *Catharus ustulatus* | - | - | **-** | 0 | 0 | **11** |
|  |  |  | Varied Thrush | *Ixoreus naevius* | - | - | **-** | 0 | 0 | **11** |
|  |  |  | Veery | *Catharus fuscescens* | - | - | **-** | 0 | 0 | **1** |
|  |  |  | Western Bluebird | *Sialia mexicana* | - | - | **-** | 0 | 0 | **1** |
|  | **Tyrannidae**  **(Tyrant Flycatchers)** |  | Alder Flycatcher | *Empidonax alnorum* | - | - | **-** | 0 | 0 | **1** |
|  | **Vireonidae**  **(Vireos, Shrike-Babblers, Erpornis)** |  | Red-eyed Vireo | *Vireo olivaceus* | - | - | **-** | 0 | 0 | **3** |
|  | **Unidentified Passeriformes** |  | Unidentified Songbird | *-* | - | - | **-** | 0 | 0 | **4** |
|  |  |  | Unidentified Sparrow | *-* | - | - | **-** | 0 | 0 | **3** |
| **Total Passeriformes** |  |  |  |  | **-** | **-** | **-** | **224 (12.9%)** | **8 (0.5%)** | **1731** |
| **Pelecaniformes** | **Ardeidae**  **(Herons, Egrets, Bitterns)** |  | American Bittern | *Botaurus lentiginosus* | - | - | **-** | 0 | 0 | **5** |
|  |  |  | Great Blue Heron | *Ardea herodias* | - | - | **-** | 10 (17.9%) | 0 | **56** |
|  |  |  | Great Egret | *Ardea alba* | - | - | **-** | 0 | 0 | **1** |
|  |  |  | Green Heron | *Butorides virescens* | - | - | **-** | 0 | 0 | **1** |
|  |  |  | Least Bittern | *Ixobrychus exilis* | - | - | **-** | 0 | 0 | **1** |
|  | **Pelecanidae**  **(Pelicans)** |  | American White Pelican | *Pelecanus erythrorhynchos* | - | - | **-** | 34 (69.4%) | 1 (2.0%) | **49** |
| **Total Pelecaniformes** |  |  |  |  | **-** | **-** | **-** | **44 (38.9%)** | **1 (0.9%)** | **113** |
| **Piciformes** | **Picidae**  **(Woodpeckers)** |  | American Three-toed Woodpecker | *Picoides dorsalis* | - | - | **-** | 0 | 0 | **1** |
|  |  |  | Downy Woodpecker | *Dryobates pubescens* | - | - | **-** | 0 | 0 | **3** |
|  |  |  | Northern Flicker | *Colaptes auratus* | - | - | **-** | 0 | 0 | **15** |
|  |  |  | Pileated Woodpecker | *Dryocopus pileatus* | - | - | **-** | 0 | 0 | **3** |
|  |  |  | Red-breasted Sapsucker | *Sphyrapicus ruber* | - | - | **-** | 0 | 0 | **1** |
|  |  |  | Yellow-bellied Sapsucker | *Sphyrapicus varius* | - | - | **-** | 0 | 0 | **2** |
|  | **Unidentified Piciformes** |  | Unidentified Woodpecker | *-* | - | - | **-** | 0 | 0 | **1** |
| **Total Piciformes** |  |  |  |  | **-** | **-** | **-** | **0** | **0** | **26** |
| **Podicipediformes** | **Podicipedidae**  **(Grebes)** |  | Eared Grebe | *Podiceps nigricollis* | - | - | **-** | 14 (73.7%) | 0 | **19** |
|  |  |  | Horned Grebe | *Podiceps auritus* | 0 | 0 | **83** | - | - | **-** |
|  |  |  | Pied-Billed Grebe | *Podilymbus podiceps* | 0 | 0 | **1** | 0 | 0 | **3** |
|  |  |  | Red-Necked Grebe | *Podiceps grisegena* | - | - | **-** | 2 (25.0%) | 0 | **8** |
|  |  |  | Western Grebe | *Aechmophorus occidentalis* | - | - | **-** | 17 (89.5%) | 0 | **19** |
|  | **Unidentified Podicipediformes** |  | Unidentified Grebe | *-* | - | - | **-** | 0 | 0 | **1** |
| **Total Podicipediformes** |  |  |  |  | **0** | **0** | **84** | **33 (66.0%)** | **0** | **50** |
| **Procellariiformes** | **Hydrobatidae (Northern Storm-Petrels)** |  | Leach's Storm-petrel | *Hydrobates leucorhous* | 0 | 0 | **231** | 0 | 1 (4.2%) | **24** |
|  | **Procellariidae (Shearwaters, Petrels)** |  | Cory's Shearwater | *Calonectris diomedea* | - | - | **-** | 0 | 0 | **12** |
|  |  |  | Great Shearwater | *Ardenna gravis* | - | - | **-** | 1 (6.7%) | 0 | **15** |
|  |  |  | Manx Shearwater | *Puffinus puffinus* | - | - | **-** | 0 | 0 | **7** |
|  |  |  | Northern Fulmar | *Fulmarus glacialis* | - | - | **-** | 1 (20.0%) | 0 | **5** |
|  |  |  | Sooty Shearwater | *Ardenna grisea* | - | - | **-** | 0 | 0 | **5** |
| **Total Procellariiformes** |  |  |  |  | **0** | **0** | **231** | **2 (2.9%)** | **1 (1.5%)** | **68** |
| **Strigiformes** | **Strigidae**  **(Owls)** |  | Barred Owl | *Strix varia* | - | - | **-** | 2 (2.3%) | 0 | **88** |
|  |  |  | Boreal Owl | *Aegolius funereus* | - | - | **-** | 0 | 0 | **12** |
|  |  |  | Burrowing Owl | *Athene cunicularia* | - | - | **-** | 0 | 0 | **5** |
|  |  |  | Eastern Screech Owl | *Megascops asio* | - | - | **-** | 0 | 0 | **14** |
|  |  |  | Great Gray Owl | *Strix nebulosa* | - | - | **-** | 1 (6.7%) | 0 | **15** |
|  |  |  | Great-horned Owl | *Bubo virginianus* | - | - | **-** | 114 (47.9%) | 1 (0.4%) | **238** |
|  |  |  | Long-eared Owl | *Asio otus* | - | - | **-** | 0 | 0 | **8** |
|  |  |  | Northern Hawk Owl | *Surnia ulula* | - | - | **-** | 0 | 0 | **1** |
|  |  |  | Northern Saw-whet Owl | *Aegolius acadicus* | - | - | **-** | 1 (3.6%) | 0 | **28** |
|  |  |  | Short-eared Owl | *Asio flammeus* | - | - | **-** | 0 | 0 | **5** |
|  |  |  | Snowy Owl | *Bubo scandiacus* | - | - | **-** | 10 (18.5%) | 1 (1.9%) | **54** |
|  | **Tytonidae**  **(Barn-Owls)** |  | Common Barn Owl | *Tyto alba* | - | - | **-** | 1 (8.3%) | 0 | **12** |
|  | **Unidentified Strigiformes** |  | Unidentified Owl | *-* | - | - | **-** | 0 | 0 | **3** |
| **Total Strigiformes** |  |  |  |  | **-** | **-** | **-** | **129 (26.7%)** | **2 (0.4%)** | **483** |
| **Suliformes** | **Phalacrocoracidae (Cormorants, Shags)** |  | Double-crested Cormorant | *Nannopterum auritum* | - | - | **-** | 42 (44.2%) | 0 | **95** |
|  |  |  | Great Cormorant | *Phalacrocorax carbo* | - | - | **-** | 0 | 0 | **3** |
|  | **Sulidae**  **(Boobies, Gannets)** |  | Northern Gannet | *Morus bassanus* | 4 (1.3%) | 0 | **321** | 122 (66.0%) | 1 (0.5%) | **185** |
| **Total Suliformes** |  |  |  |  | **4 (1.3%)** | **0** | **321** | **164 (58.0%)** | **1 (0.4%)** | **283** |
| **Grand Total** |  |  |  |  | **586 (5.2%)** | **1160 (10.3%)** | **11295** | **1710 (27.4%)** | **62 (1.0%)** | **6246** |

^a^ Unless otherwise indicated, species that tested positive for HPAIV based on pooled swab samples from sick and dead wild bird surveillance were generally found to have lesions consistent with HPAIV infection upon gross and histologic examination. However, the proportion of birds that underwent gross and histologic examination varied by province/territory and in some cases, this information was not available. In at least one province/territory, no birds that tested positive for AIV underwent gross or histologic examination. In another province/territory, 76% of birds that were suspect or confirmed positive for HPAIV underwent gross and histologic examination of which 97% were interpreted by the pathologist to have died as a result of HPAIV.

^b^ Two of the Rock Pigeons did not have lesions consistent with HPAIV on gross and histologic examination. Cause of death in both of these cases was determined to be avitrol poisoning. The third Rock Pigeon did not undergo histologic examination, but gross lesions included: congestion (moderate to marked) predominantly in the lungs, heart, and brain, in good nutritional condition (moderate fat stores) and no signs of trauma.

^c^ One Wild Turkey did not have lesions consistent with HPAIV on gross and histologic examination. Cause of death was determined to be trauma. Gross and histologic results not available on the second wild turkey.

^d^ The House Sparrow did not have lesions consistent with HPAIV on gross and histologic examination. Cause of death in this case was determined to be drowning.

Table S2. Acknowledgements of collaborators that have contributed to the collection and curation of these data.

|  | Name | Affiliation |
| --- | --- | --- |
| Federal | Andrew Kennedy  Sabina Wilhelm  Carina Gjerdrum  Scott Gilliland  Sarah Wong  Bruce Pollard  Matthew English  Andrew Hicks  Ted Barney  Chris Ward  Julie Paquette  Pierre Ryan  Regina Wells  Becky Whittam | Canadian Wildlife Service, Atlantic Region, Environment and Climate Change Canada |
|  | Francis St-Pierre  Mathieu Tétreault  Yannick Seyer  Jean-François Rail | Canadian Wildlife Service, Quebec Region, Environment and Climate Change Canada |
|  | Brigitte Collins  Denby Sadler  Ross Wood  Shawn Meyer | Canadian Wildlife Service, Ontario Region, Environment and Climate Change Canada |
|  | Mark Schuster  Blake Bartzen  Keith Warner  Owen Andrushuk  Darin Walker  Pat Bergen  Ferguson Moore | Canadian Wildlife Service Aquatic Unit, Prairie Region, Environment and Climate Change Canada |
|  | William O’Shea  Xiao Jun (Jim) Song  Jacob Hubner | Canadian Wildlife Service, Pacific Region, Environment and Climate Change Canada |
|  | Ray Alisauskas  Jamille McLeod  Landon McPhee  Alana Weber  Karen Gesy  Josh Cunningham | Science and Technology Branch, Environment and Climate Change Canada |
|  | Julie Pare  Noel Harrington  NCFAD avian diseases, genomics and sample receiving unit staff | Canadian Food Inspection Agency |
|  | Dave McRuer and staff | Atlantic and Quebec Field Units, Parks Canada |
| Provincial/Territorial | Garry Gregory  Matt Ginns  Ross Bernard  and other staff | Government of Prince Edward Island, Department of Environment, Water and Climate Change |
|  | Beverly Dawe  Blair Adams  Chuck Porter  Tina Leonard  and other staff | Government of Newfoundland and Labrador, Department of Fisheries, Forestry and Agriculture, Forestry and Wildlife Branch |
|  | Lee Millett  All DNRR Regional Services staff involved in incident responses and wildlife specimen collections | Government of Nova Scotia, Department of Natural Resources and Renewables |
|  | Staff | Government of New Brunswick, Department of Natural Resources and Energy Development |
|  | Staff | Government of New Brunswick, Department of Agriculture, Aquaculture, and Fisheries |
|  | Kelsey Saboraki | Manitoba Department of Natural Resources and Northern Development, Wildlife Branch |
|  | Tracy Scammell-Lafleur  Virology Section Staff | Manitoba Department of Agriculture, Veterinary Diagnostic Services |
|  | Staff of the reporting center  Staff of the Laboratoire de santé animale de Saint-Hyacinthe | Ministère de l’Agriculture, des Pêcheries et de l’Alimentation du Québec |
|  | Frédérick Lelièvre  Christine Thibault  Wildlife officers, biologists, and technicians in regions | Ministère de l’Environnement, de la Lutte contre les changements climatiques, de la Faune et des Parcs du Québec |
|  | Ayden McGuire Sherritt  Kim Bennett  Airboat Waterfowl Banding Team | Ontario Ministry of Natural Resources and Forestry, Wildlife Research and Monitoring Section |
|  | Maud Henaff  Kristenn Magnusson  Michelle Thompson  Mary VanderKop | Animal Health Unit, Environment Yukon |
|  | Kandis Villebrun | Government of Northwest Territories, Department of Environment and Climate Change |
|  | Sammy Angnaluak Monica Angohiatok Johanne Coutu-Autut Erik Ikoe Desmond Inaksajak Alexander Kadlutsiak Peter Kattegatsiak Brad McInnes Jonah Qittusuk James Simonee Jack Skillings Candice Sudlovenick Kevin Sudlovenick Russell Toolooktook | Wildlife Operations Division, Department of Environment, Government of Nunavut |
| Academic, Other | Robyn MacPhee  Sarah Ogilvie | AVC Diagnostic Services, Atlantic Veterinary College, University of Prince Edward Island, Charlottetown, Prince Edward Island |
|  | Staff | Canadian Wildlife Health Cooperative, National Office |
|  | Viviane Casaubon  Judith Viau  Émilie L. Couture  Kathleen Brown  Shannon Ferrell  Ariane Guertin-Cabana | Canadian Wildlife Health Cooperative, Québec |
|  | Magella Guillemette | Université du Québec à Rimouski |
|  | Jean-Francois Giroux | Université du Québec à Montréal |
|  | Pierre Legagneux | Université Laval |
|  | Lenny Shirose  Laura Dougherty  Communication Team | Canadian Wildlife Health Cooperative, Ontario-Nunavut |
|  | Mitch Weegman | University of Saskatchewan |
|  | Erin Moffat | Canadian Wildlife Health Cooperative, Western Northern Region |
