# Supplementary material for: Avian influenza viruses in wild birds in Canada following incursions of highly pathogenic H5N1 virus from Eurasia in 2021–2022
Source: mBio. 2024 Jul 16;15(8):e03203-23. doi: 10.1128/mbio.03203-23 (PMC11323545; doi:10.1128/mbio.03203-23)
Supplement: Supplemental Figures — Figures S1 to S5. [file mbio.03203-23-s0005.docx]

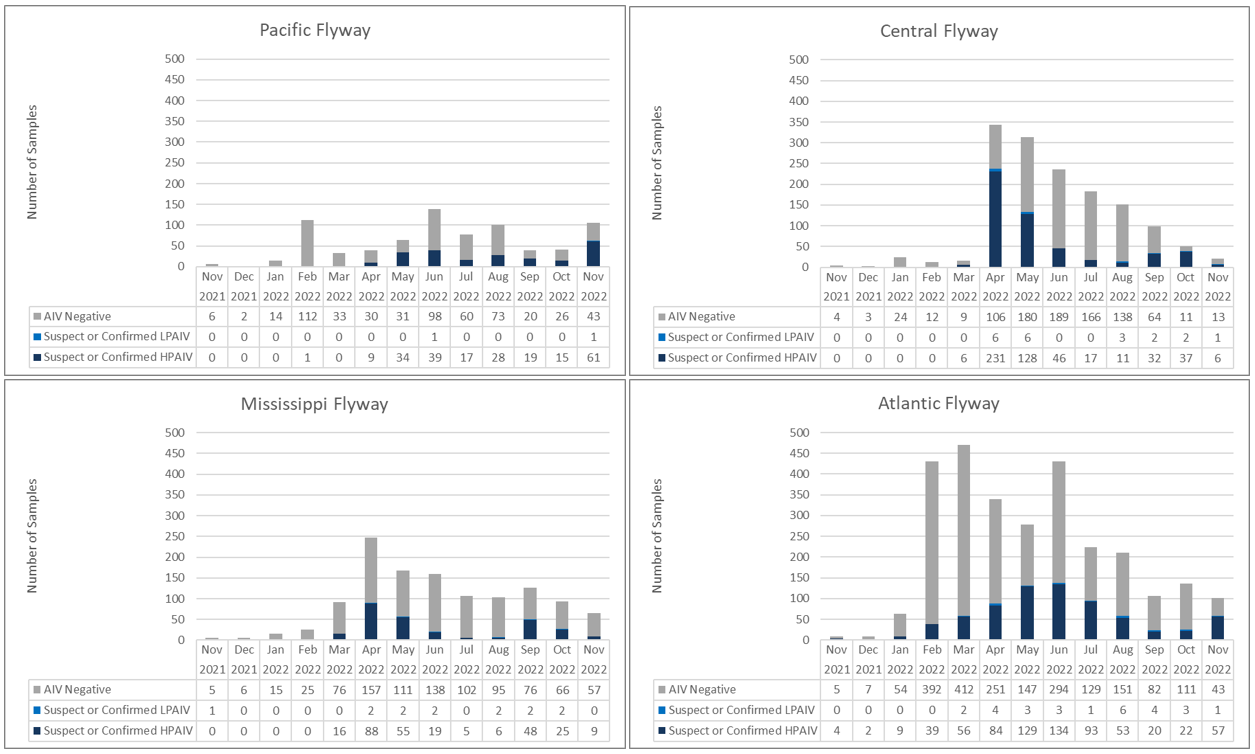
**Supplemental Figures**

Fig. S1. Sick and dead wild birds tested for avian influenza virus (AIV) monthly between November 2021 and November 2022 across Canada stratified by flyway.


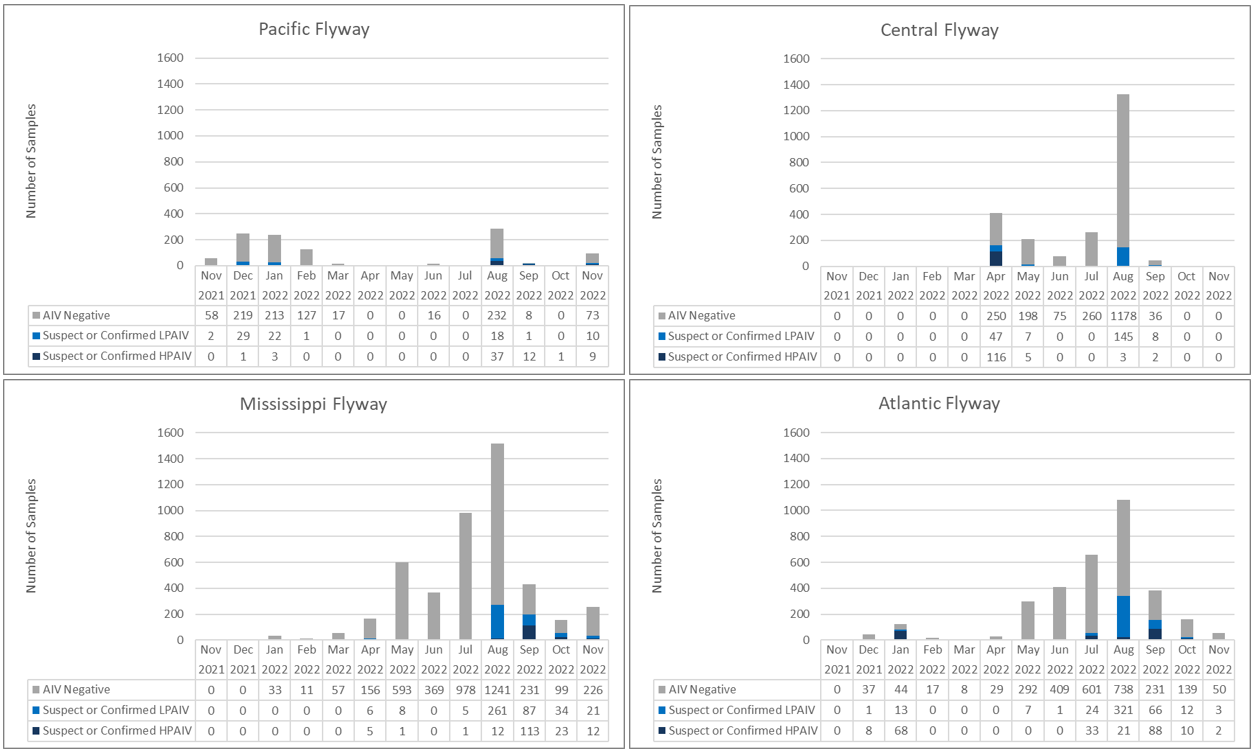
Fig. S2. Live and hunter-harvested wild birds tested for avian influenza virus (AIV) monthly between November 2021 and November 2022 across Canada stratified by flyway.


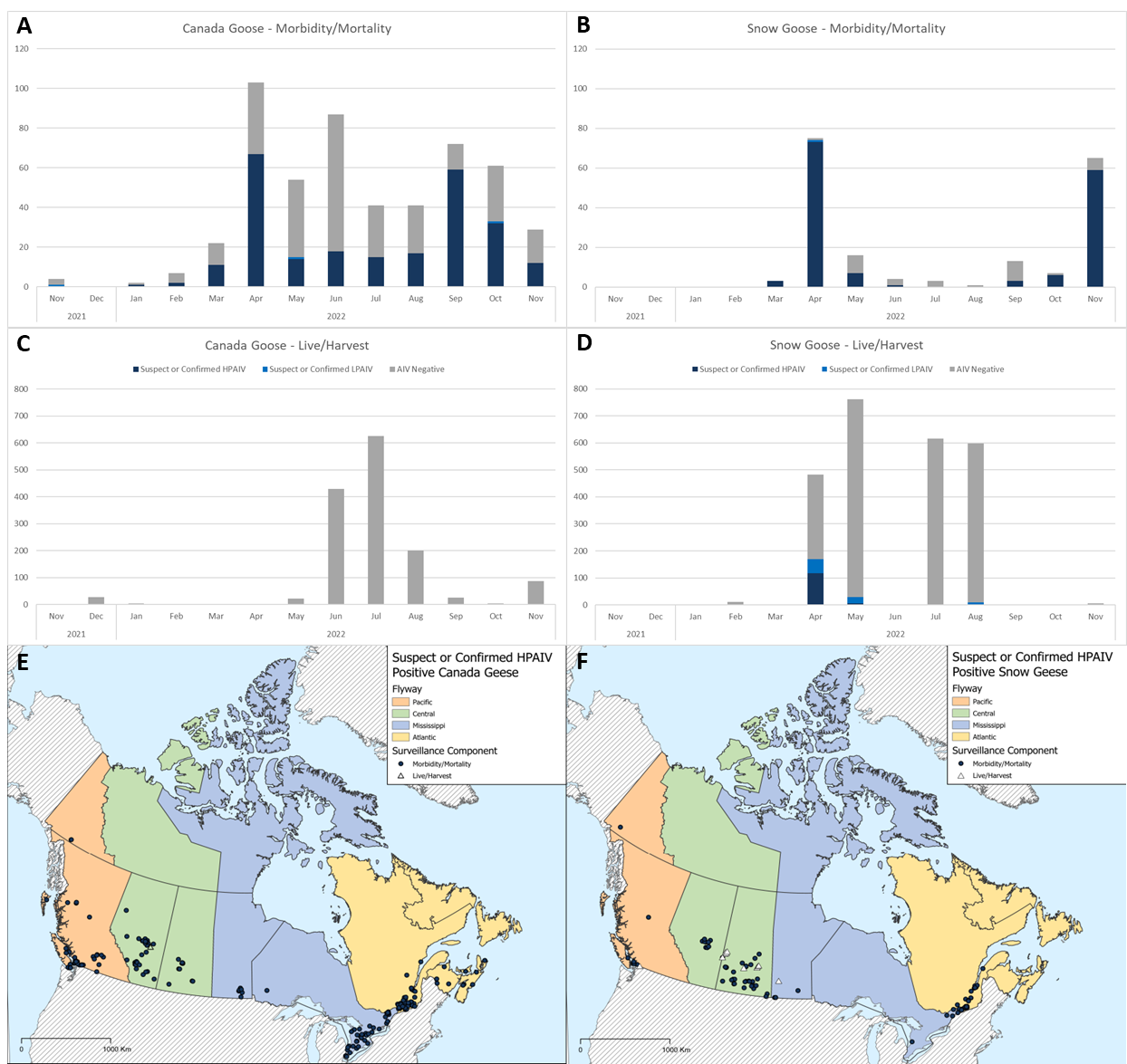


Fig. S3. Canada Geese (A, C) and Snow Geese (B, D) tested for avian influenza virus (AIV) monthly between November 2021 and November 2022 across Canada stratified by surveillance component (i.e., sick/dead and live/hunter harvest). The location of suspect and confirmed highly pathogenic avian influenza virus (HPAIV) positive Canada Geese € and Snow Geese (F).

Fig. S4. Dabbling ducks (A, C) and diving ducks and seaducks (B, D) tested for avian influenza virus (AIV) monthly between November 2021 and November 2022 across Canada stratified by surveillance component (i.e., sick/dead and live/hunter harvest). The location of suspect and confirmed highly pathogenic avian influenza positive dabbling ducks (E) and diving ducks and seaducks (F).


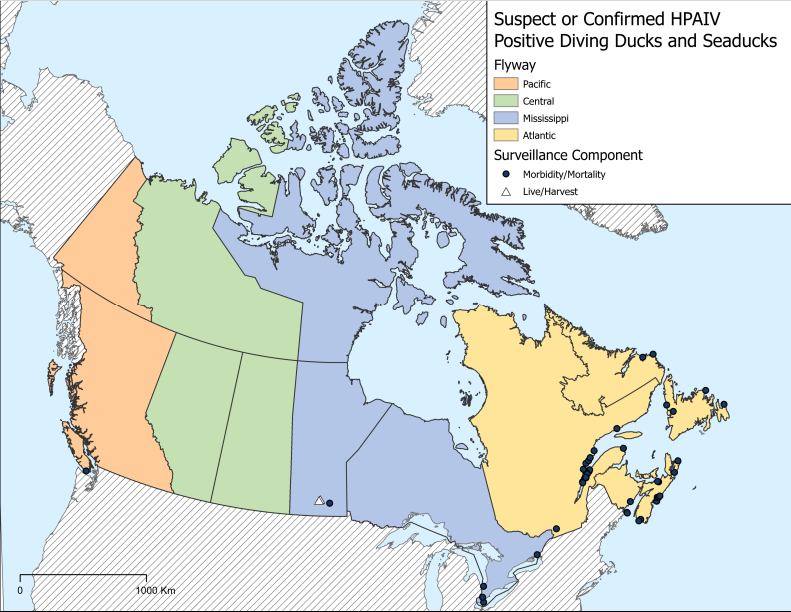

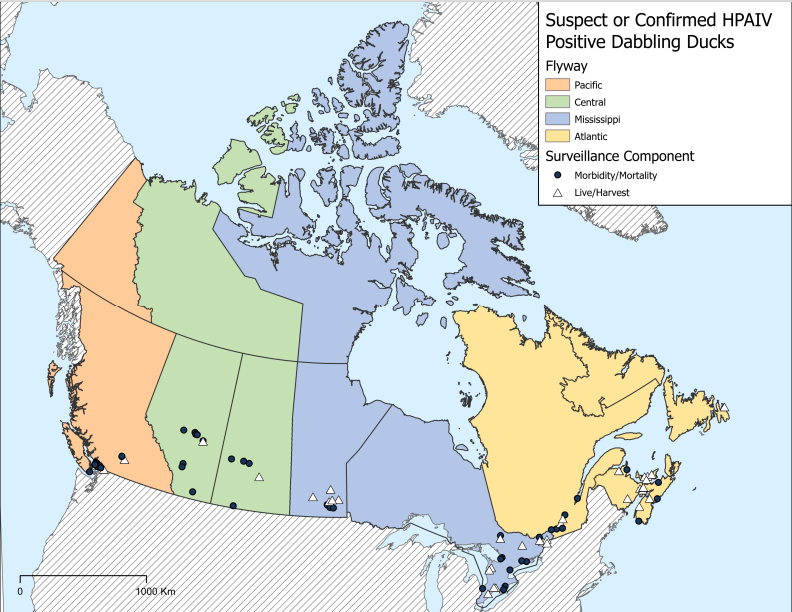

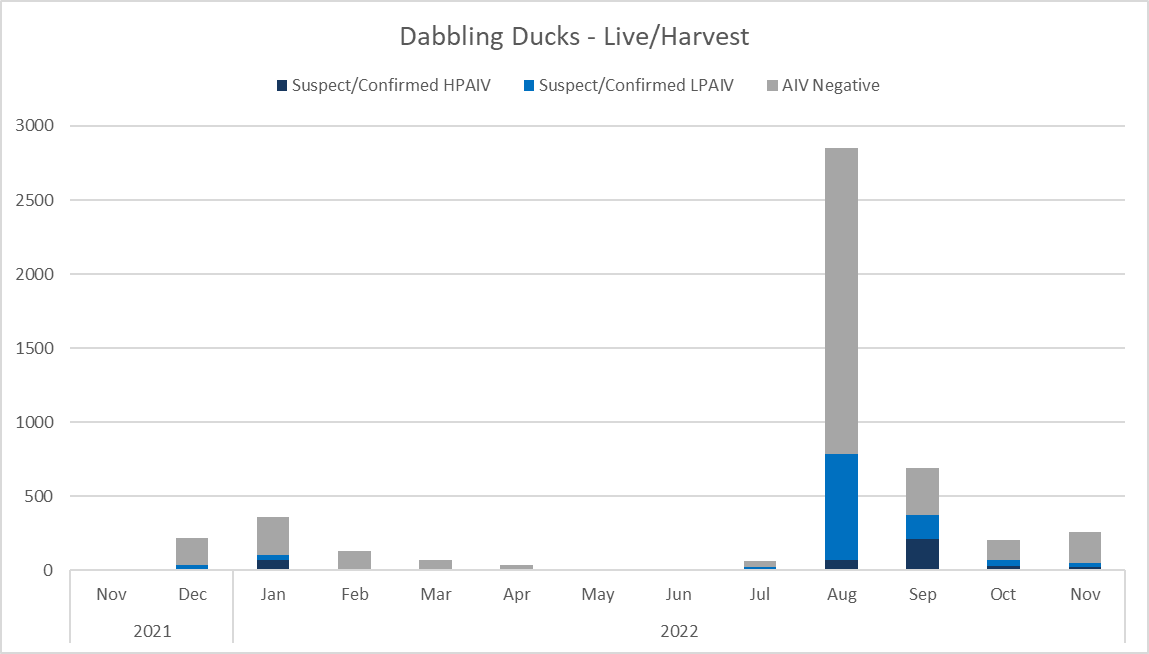

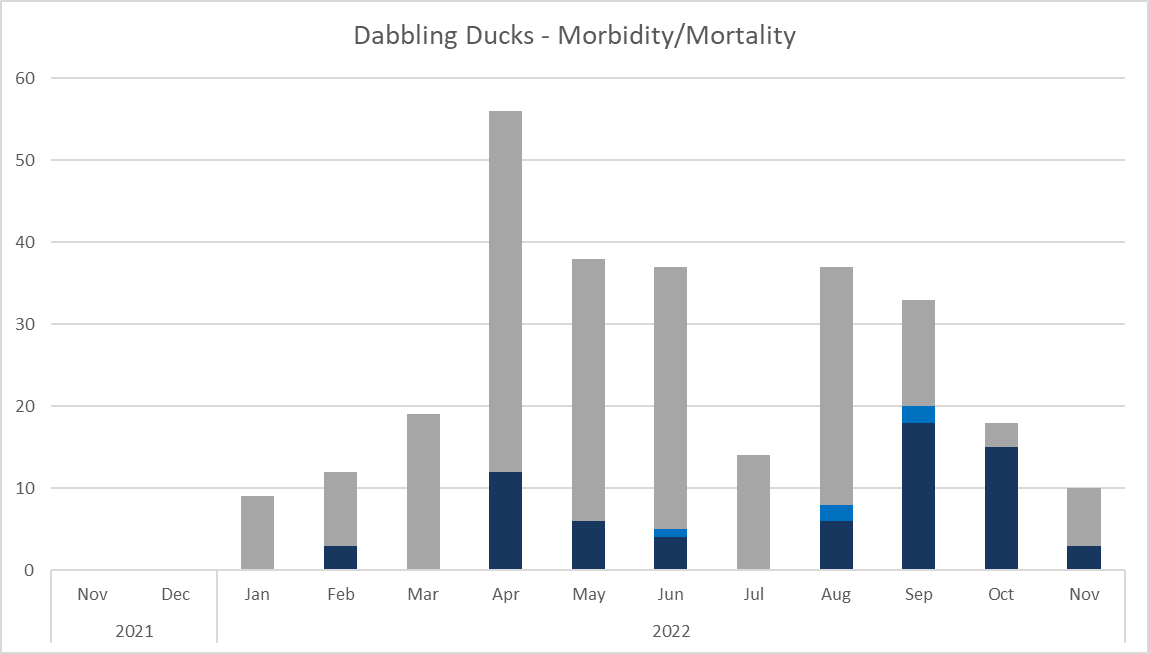

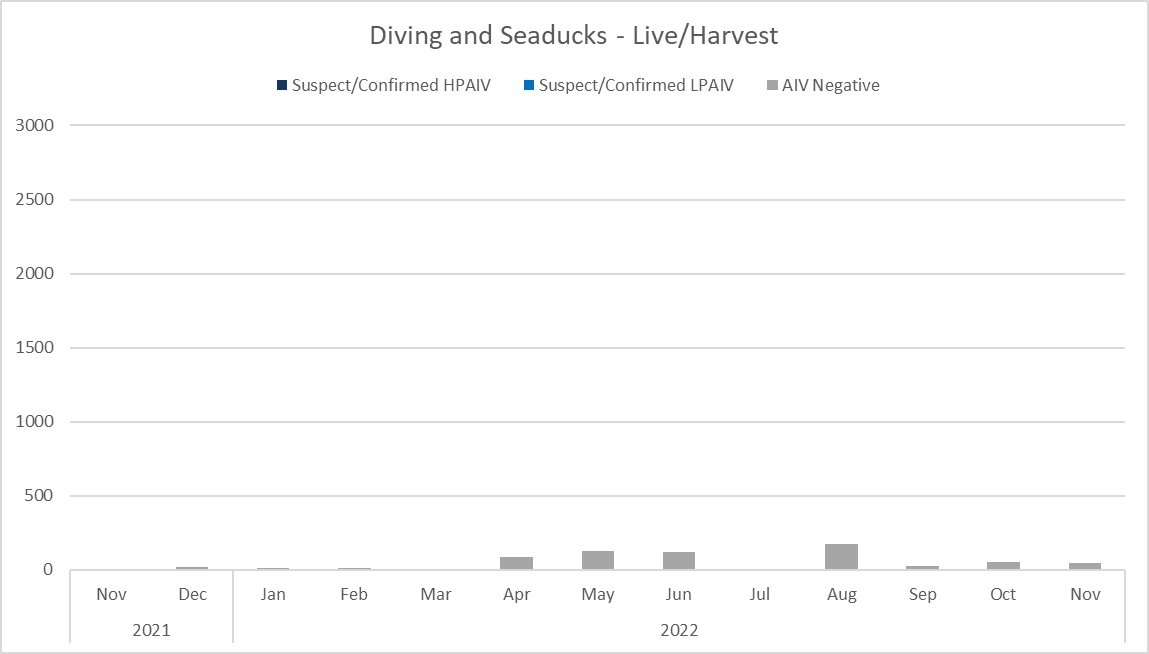

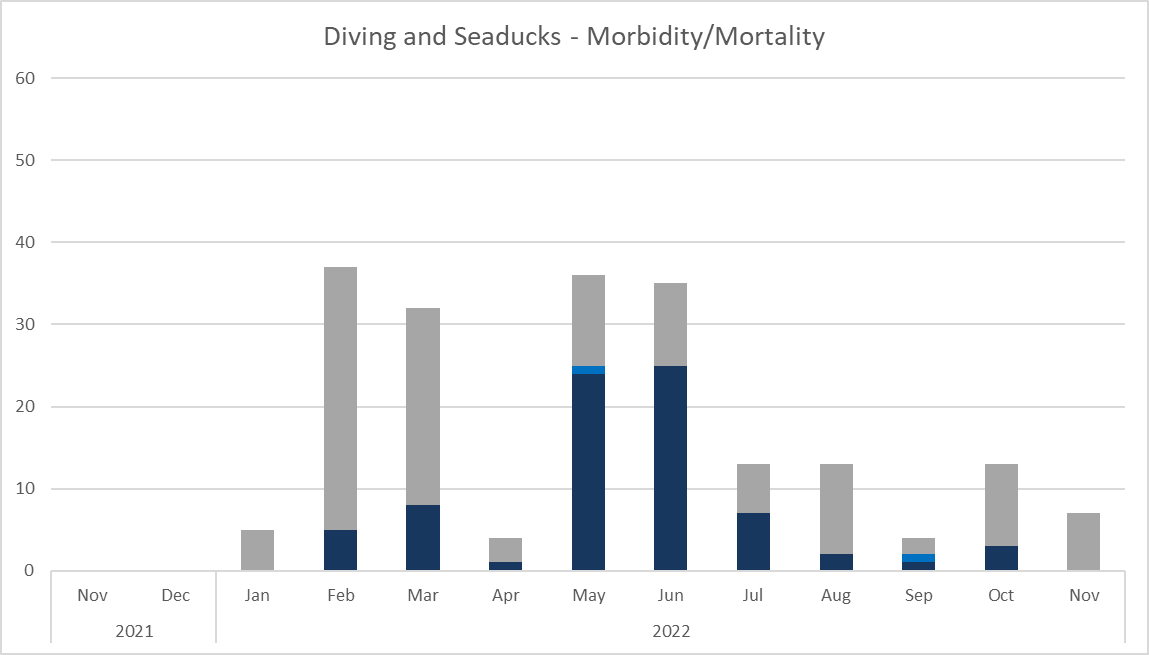


**A**

**C**

**E**

**B**

**D**

**F**


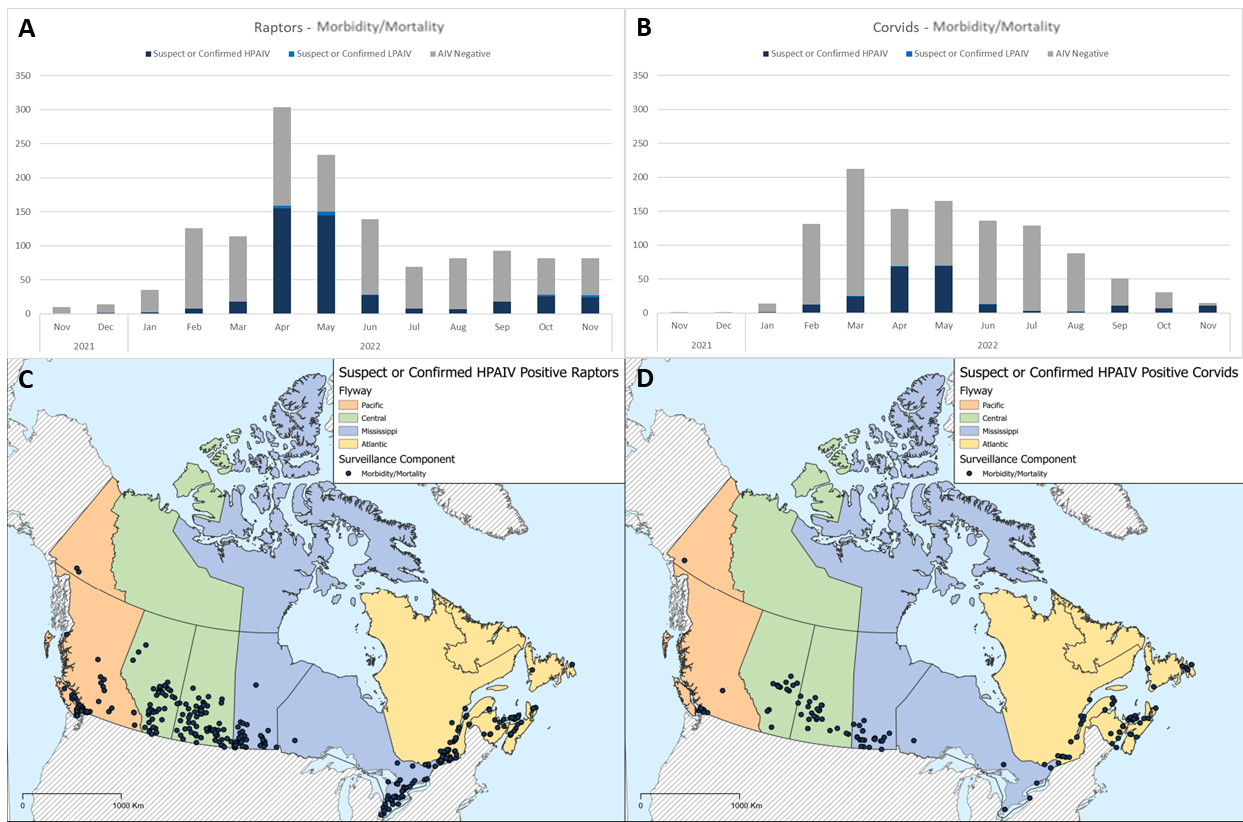


Fig. S5. Raptors (A) and corvids (B) tested for avian influenza virus (AIV) monthly between November 2021 and November 2022 across Canada through morbidity/mortality surveillance. The location of suspect and confirmed highly pathogenic avian influenza positive raptors (C) and corvids (D).
